# Health-related quality of life and utility values among patients with anxiety and/or depression in a low-income tertiary care setting: a cross-sectional analysis

**DOI:** 10.1007/s11136-024-03735-8

**Published:** 2024-07-16

**Authors:** Yared Belete Belay, Cathrine Mihalopoulos, Yong Yi Lee, Lidia Engel

**Affiliations:** 1 School of Public Health and Preventive Medicine, Monash University, Melbourne, Australia; 2 School of Pharmacy, Mekelle University, Mekelle, Ethiopia; 3 School of Public Health, The University of Queensland, Brisbane, Australia; 4 Queensland Centre for Mental Health Research, Brisbane, Australia

**Keywords:** Anxiety, Depression, EQ-5D-5L, Health-related quality of life, Health state utility values

## Abstract

**Objectives:**

To describe the health-related quality of life (HRQoL), estimate the associated health state utility values (HSUVs) and explore factors associated with HRQoL of patients with anxiety and/or depression in a resource-limited hospital setting.

**Methods:**

A cross-sectional survey involving 462 participants was conducted in a hospital setting. The Amharic version of the EQ-5D-5 L assessed HRQoL, while the GAD-7 and PHQ-9 measured severity of anxiety and depression symptoms respectively. HSUVs were analysed based on clinical and demographic profiles; mean differences were compared using t-tests and one-way ANOVA; Scheffe’s post hoc comparisons and effect sizes (Cohen’s d statistic) were used to assess the magnitude of group differences. Factors associated with HRQoL were explored using regression analysis.

**Results:**

The mean HSUV was 0.87 (SD = 0.17) and the EQ VAS was 71.4 (SD = 19.1). Patients with both anxiety and depression scored significantly lower (HSUV = 0.83 [0.16], EQ VAS = 64.4 [17.9]) compared to those with either anxiety only (HSUV = 0.88 [0.17], EQ VAS = 75.3 [17.9]) or depression only (HSUV = 0.89 [0.18], EQ VAS 74.4 [19.7]). Males had slightly higher mean scores than females, while those aged 18–35 years demonstrated the highest scores on both the EQ-5D-5 L and EQ VAS. Older age (β=-0.002), higher PHQ-9 scores (β=-0.008) and comorbid hypertension (β=-0.07) associated with lower HSUVs. Lower EQ VAS scores were associated with being female (β=-4.4), having comorbid hypertension (β=-7.4) and higher PHQ-9 scores (β=-0.86), while a positive association was found with having ‘more than enough’ income (β = 11.8).

**Conclusions:**

Older age, severity or co-diagnosis of anxiety or depression and comorbid conditions were associated with lower HRQoL, highlighting the need for better interventions to improve the HRQoL of patients with anxiety and depression in Ethiopia.

**Supplementary Information:**

The online version contains supplementary material available at 10.1007/s11136-024-03735-8.

## Introduction

Over the past decade, there has been a 13% increase in mental health conditions globally, with one in five years of disability attributed to mental health issues [1]. Before the COVID-19 pandemic, global anxiety disorder prevalence was 3,824.9 per 100,000 population (298 million people), rising to 4802.4 (374 million people) post-pandemic, indicating an additional 76.2 million cases (25.6% increase). Similarly, for major depressive disorder, pre-pandemic prevalence was 2470.5 cases per 100,000 (193 million people), increasing to 3152.9 (246 million people) post-pandemic, with an additional 53.2 million cases (27.6% increase) [2]. Low- and middle-income countries (LMICs) have been disproportionately impacted by this health problem as evidenced by highest age standardised prevalence [3]. Globally, mental healthcare is underfunded and underprovided, with less than 2% of government health expenditures being allocated to mental health [1]. This is in stark contrast to the economic burden imposed by disorders such as depression and anxiety, which costs the global economy USD 1 trillion [1].

Anxiety and depression can significantly impact individuals’ health-related quality of life (HRQoL) [4], which is usually defined as a subjective assessment of an individual’s physical, emotional and social well-being [5]. HRQoL measures have gained increasing importance as an outcome measure to provide a comprehensive evaluation of the impact of illness on patients’ lives [6]. One of the challenges in assessing the impact of health interventions or programs on patient outcomes is the need to convert subjective responses about HRQoL into quantifiable measures that can be compared across different interventions or populations. Methods to measure ‘utility values’ that denote the relative value of different levels of HRQoL have been developed to address this challenge [7].

Utilities are used to calculate the quality-adjusted life year (QALY) metric, which is a commonly used outcome measure in cost-utility analysis (CUA) [8]. The QALY combines extensions to a person’s life expectancy and their HRQoL into a single metric. It can be used to evaluate health outcomes produced by different interventions and services across a variety of diseases and population groups. The length of life is adjusted using a utility value (a.k.a., utility weight) that numerically represents HRQoL on a scale between 0 (denoting death) and 1 (denoting perfect health). The most commonly used approach for estimating utility values is through the use of preference-based HRQoL instruments that are conventionally referred to as multi-attribute utility instrument (MAUI). MAUIs ask respondents to rate their health across several dimensions (i.e., their health state). Preference weights are then applied using a pre-determined scoring algorithm to estimate the corresponding utility value [7, 9]. Utility values are an important outcome measure and can help estimate the health benefits of different interventions and treatments [10]. To this end, several generic and disease-specific tools have been developed to measure HRQoL and calculate utility weights [11]. One of the most widely used and accepted measures for establishing utility weights is the EuroQol − 5 dimension (EQ-5D) [12]. The EQ-5D is a standardised questionnaire that measures health status across five dimensions on a three-level or five-level response scales: Mobility (Mob), Self-care (SC), usual activities (U/A), pain/discomfort (P/D), and anxiety/depression (A/D). It has value sets that have been validated in multiple countries and is available in over 200 languages. The tool contains the EQ-5D descriptive system and the EQ visual analogue scale (EQ VAS) [13].

In many resource-constrained settings, healthcare decision-making requires evidence from cost-effectiveness analyses to facilitate the efficient allocation of limited resources across competing healthcare interventions. Utility values are important elements in both trial- and model-based economic evaluations that use QALYs to guide resource allocation decisions [7]. Within the context of anxiety and depression, previous studies have provided such utility values for different disease severity levels [14]. However, existing utility values have been largely derived from developed countries, limiting their applicability for modelled economic studies conducted for LMICs [15]. Further, given that utilities can be derived using different HRQoL questionnaires that produce different scores, it is imperative to estimate utility values for different severity levels using the same instrument to inform modelled economic analysis [16, 17]. To address these gaps, the aim of this study was to describe the HRQoL, estimate the associated health state utility values (HSUVs) and explore factors associated with HRQoL of patients with anxiety and/or depression in a resource-limited hospital setting.

## Method

### Study design

This cross-sectional study, conducted in a tertiary care mental health hospital, recruited patients with anxiety and/or depression. The study followed the Strengthening the Reporting of Observational Studies in Epidemiology (STROBE) guidelines to ensure accurate and complete reporting of the study’s methods and results [18]. A copy of the completed STROBE checklist for this study is available in Supplementary File [Supplementary file 1].

### Study setting and recruitment

This hospital-based survey recruited participants who were receiving outpatient services at Amanuel Mental Specialised Hospital. It is the only specialised psychiatric hospital in Ethiopia, situated in Addis Ababa, the country’s capital. Patients from all regions of the country, both urban and rural, visit the hospital for inpatient and outpatient psychiatric care. This ensured the inclusion of participants with diverse demographic backgrounds, making the sample more representative. This study involved patients with anxiety and/or depression who attended outpatient follow-up visits at the hospital. Patients treated in an inpatient setting were excluded from this study. Diagnosis of anxiety and depression was based on clinician assessments reported in the patient’s medical records (patient case notes). In the hospital, clinicians primarily use The Diagnostic and Statistical Manual of Mental Disorders (DSM-5) to diagnose and confirm cases of anxiety and depression [19]. After completing their outpatient visit, research nurses who had access to patients’ medical records invited eligible patients to an interview, which was done in a separate room where clinicians or other hospital staff were not involved. Eligible patients with anxiety and/or depression were asked to provide an informed consent before the interview. Further information about recruitment procedure is provided in previous publication (Belay et al., 2023) [20]. The sample size (462) was determined using a single proportion formula, considering a 95% confidence level, 5% margin of error, a proportion (P) of 0.5 and a 20% non-response rate [21]. Participants were consecutively recruited using a convenience sampling method until the target sample size was reached, comprising approximately equal thirds: 149 with anxiety, 159 with depression, and 154 with both anxiety and depression.

### Data collection and instrument

The data collection was carried out between June and October 2021 by five research nurses using an interviewer-administered approach to ensure the participation of individuals with no formal education who might struggle with self-complete tasks. The Amharic version of the interviewer-administered EQ-5D-5 L, which is widely used as the official working language in Ethiopia, was employed to assess HRQoL. This translation, conducted as part of the Ethiopian valuation study, followed the standardised approach recommended by the EuroQol Group [22, 23]. Validated Amharic versions of instruments were used to collect data on participant characteristics and mental health status. This included the Patient Health Questionnaire-9 (PHQ-9), a nine-item questionnaire rating depressive symptoms in the last two weeks on a scale of zero (not at all) to 4 (nearly every day) [24, 25], and the Generalized Anxiety Disorder-7 (GAD-7), seven-item questionnaire rating anxiety symptoms on a scale of zero (not at all) to 4 (nearly every day) in the last two weeks [26, 27]. All HRQoL measures, participants’ demographic (gender, age, education, employment status, income, and marital status), behavioural characteristics (smoking and alcohol) and symptom severity (PHQ-9 and GAD-7), were self-reported by patients. Patient clinical profiles (diagnosis, type of anxiety or depression, duration of illness, comorbidity, and medication) were collected from patient medical records (patient case notes) that had been documented by clinicians.

### Analysis

The analysis incorporated data from all 462 participants, with no missing responses, due to the data collection approach using in person interviews. Demographic and clinical characteristics were described using frequency and percentage. The EQ-5D-5 L responses were converted into a HSUVs using value set developed for Ethiopia [23]. To derive the HSUVs value from each EQ-5D-5 L health profile, we applied a formula that subtracts the disutility weight of each level in each dimension from 1 (representing full health). This approach is outlined in the methods for analysing and reporting EQ-5D data by Devlin et al.,2020 [28].

The study presented the proportions of reported problems (level 2 to level 5) for each dimension of the EQ-5D-5 L across demographic and clinical characteristics of the population. The PHQ-9 scores were categorised for depression severity: 1–4 (minimal), 5–9 (mild), 10–14 (moderate), 15–19 (moderately severe), and 20–27 (severe) [29]. GAD-7 scores were categorised for anxiety severity: 0–4 (minimal), 5–9 (mild), 10–14 (moderate), and 15–21 (severe) [27]. Internal consistency was assessed using Cronbach’s alpha and was considered acceptable only if it exceeded 0.70.

HSUVs by severity level and other clinical and demographic profile were described using means and standard deviations (SD). T-test and one-way ANOVA analyses were used to compare the mean difference in HSUVs across different groups. Scheffe’s post hoc comparisons and effect sizes (Cohen’s d statistic) were used to assess the magnitude of group differences. Cohen’s d used to quantify differences by converting the mean difference between two groups into standard deviation units (mean of group 1 - mean of group 2/ SD). Cohen’s d scores were interpreted as follows: < 0.2 = small effect size, approximately 0.5 = moderate effect size, and > 0.80 = large effect size [30]. To explore factors associated with HRQoL, the univariate association between independent variables and HRQoL measures (HSUV and EQ VAS) was examined using ordinary least squares regression. A hierarchical multiple regression analysis was then conducted to determine the relationship between demographic variables, clinical variables and behavioural variables with HSUVs and EQ VAS. In step 1, a regression model was constructed to evaluate the relationship between HSUVs or EQ VAS scores and demographic variables. In step 2, the analysis was extended to include clinical variables and in step 3, behavioural variables were added to the model. Model selection was performed based on the adjusted R-squared, Akaike information criterion (AIC), and Bayesian information criterion (BIC). The model with the highest adjusted R-squared and the lowest AIC and BIC was considered the best-fitting model. Model 3 demonstrated better performance based on the performance indices of the hierarchical regression analysis. The significance level was set at *p* < 0.05. The analysis was performed using Stata version 17.

## Result

### Participant characteristics

We recruited 462 participants with comparable numbers of patients diagnosed with anxiety only (149), depression only (159) and comorbid anxiety and depression (154). The most commonly reported types of anxiety and depression was generalised anxiety disorders (56.1%) and major depressive disorder (64.7%) respectively. Most participants (53.9%) were below the age of 35 years, 45.2% were unmarried, 30.7% had primary education, 66.7% reported insufficient income, 79.7% did not smoke and 61.4% reported having never consumed alcohol. According to the PHQ-9 scores, 33.6% of respondents had moderate depression, with the rest distributed among mild (23.5%), moderately severe (21.0%), and severe (21.8%) depression. Based on the GAD-7 scores 37.5% of participants reported mild anxiety, 40.0% had moderate, and 22.5% reported severe anxiety (See Tables 1 and 2).

**Table 1:** Summary score of HSUVs and EQ VAS by socio-demographic characteristics (*n* = 462)

Demographic profiles		n (%)	EQ-5D-5 L			EQ VAS		
			Mean (SD)	*p-value*	*Scheffe p-value; Cohen’s d*	Mean (SD)	*p-value*	*Scheffe p-value; Cohen’s d*
Gender	Male (1)	231 (50)	0.88 (0.17)	0.27	0.11	73.1 (19.1)	0.05	0.18
	Female (2)	231 (50)	0.86 (0.18)			69.6 (19.1)		
Age (in years)	18–35 (1)	249 (53.9)	0.90 (0.14)	< 0.001	1vs2 (0.013;0.30) ^*^ 1vs3 (0.002;0.83) ^*^ 2vs3 (0.142;0.35)	73.8 (17.5)	0.001	1vs2 (0.072;0.22) 1vs3 (0.005;0.67) ^*^ 2vs3 (0.115;0.38)
	36–55 (2)	183 (39.6)	0.85 (0.20)			69.6 (20.2)		
	> 55 (3)	30 (6.5)	0.78 (0.18)			61.8 (22)		
Marital status	Unmarried (1)	209 (45.2)	0.88 (0.17)	0.10	1vs2 (0.917;0.60) 1vs3 (137;0.35) 1vs4 (0.710;0.23) 2vs3 (0.343;0.30) 2vs4 (0.871;0.18) 3vs4 (0.989; -0.10)	73.4 (18.8)	0.004	1vs2 (0.943;0.06) 1vs3 (0.020;0.46) ^*^ 1vs4 (0.190;0.49) 2vs3 (0.071;0.41) 2vs4 (0.316;0.44) 3vs4 (1;0.02)
	Married (2)	174 (37.7)	0.87 (0.16)			72.2 (18.3)		
	Divorced (3)	57 (12.3)	0.82 (0.18)			64.5 (20.4)		
	Widowed (4)	22 (4.8)	0.84 (0.24)			64.1 (20.5)		
Education	No formal education (1)	52 (11.3)	0.82 (0.19)	0.13	1vs2 (0.474; -0.01) 1vs3 (0.303; -0.01) 1vs4 (0.430; -0.01) 1vs5 (0.985; -0.01) 2vs3 (0.995; -0.06) 2vs4 (0.998; -0.06) 2vs5 (0.813;0.24) 3vs4 (1;0) 3vs5 (0.630;0.30) 4vs5 (0.740;0.30)	67.5 (19.9)	0.17	1vs2 (0.984; -0.09) 1vs3 (0.537; -0.29) 1vs4 (0.781; -0.26) 1vs5 (0.432; -0.36) 2vs3 (0.650; -0.19) 2vs4 (0.916; -0.15) 2vs5 (0.542; -0.26) 3vs4 (0.999;0.04) 3vs5 (0.992; -0.08) 4vs5 (0.974; -0.13)
	Primary education (2)	142 (30.7)	0.88 (0.16)			69.4 (20.2)		
	Secondary education (3)	136 (29.4)	0.89 (0.16)			73 (18.5)		
	TVT /diploma^a^ (4)	69 (14.9)	0.89 (0.15)			72.2 (17.1)		
	Degree and above (5)	63 (13.6)	0.84 (0.22)			74.5 (19.2)		
Occupation	Employed (1)	227 (49.1)	0.88 (0.16)	0.03	1vs2 (0.102;0.78) 1vs3 (0.999; -0.25) 1vs4 (0.779;0.24) 1vs5 (0.992; -0.06) 2vs3 (0.101; -0.01) 2vs4 (0.422; -0.46) 2vs5 (0.085; -0.72) 3vs4 (0.761;0.01) 3vs5 (1; -0.01) 4vs5 (0.689; -0.01)	71.3 (18.6)	0.17	1vs2 (0.959;0.22) 1vs3 (01;0.-0.016) 1vs4 (0.746;0.19) 1vs5 (0.650; -0.22) 2vs3 (0.957; -0.23) 2vs4 (1; -0.02) 2vs5 (0.705; -0.43) 3vs4 (0.809;0.20) 3vs5 (0.831; -0.20) 4vs5 (0.223; -0.39)
	Pensioner (2)	14 (3)	075 (0.26)			67.1 (23.3)		
	Student (3)	81 (17.5)	0.88 (0.17)			71.6 (18.5)		
	Looking after home or family (4)	65 (14.1)	0.84 (0.18)			67.6 (21.4)		
	Others^b^ (5)	75 (16.2)	0.89 (0.18)			75.3 (18.2)		
Income	Not enough (1)	308 (66.7)	0.88 (0.16)	0.06	1vs2 (0.070;0.24) 1vs3 (0.639;0.67) 2vs3 (0.978; -0.05)	70.7 (19.1)	0.05	1vs2 (0.976; -0.03) 1vs3 (0.050; -0.51) 2vs3 (0.085; -0.49)
	Just enough (2)	129 (27.9)	0.84 (0.18)			71.2 (19)		
	More than enough (3)	25 (5.4)	0.85 (0.24)			80.4 (18.2)		
Smoking habit	Daily (1)	32 (6.9)	0.79 (0.27)	0.01	1vs2 (0.069; -0.40) 1vs3 (0.015; -0.53) ^*^ 2vs3 (0.973; -0.06)	62.5 (21.8)	0.02	1vs2 (0.112; -0.41) 1vs3 (0.022; -0.53) ^*^ 2vs3 (0.924; -0.03)
	Less than daily (2)	62 (13.4)	0.87 (0.15)			71.1 (22.4)		
	Not at all (3)	368 (79.7)	0.88 (0.16)			72.2 (18.1)		
Alcohol taking habit	Never (1)	283 (61.4)	0.89 (0.16)	< 0.001	1vs2 (0.822; -0.14) 1vs3 (0.403;0.36) 1vs4 (0.016;0.64) ^*^ 1vs5 (0.006;1.33) ^*^ 2vs3 (0.158;0.57) 2vs4 (0.005;0.85) ^*^ 2vs5 (0.002;1.91) ^*^ 3vs4 (0.689;0.24) 3vs5 (0.106;0.77) 4vs5 (0.513;0.44)	74.2 (17.8)	< 0.001	1vs2 (0.956; -0.10) 1vs3 (< 0.001; 0.71) ^*^ 1vs4 (< 0.001;0.83) ^*^ 1vs5 (0.040;1.13) ^*^ 2vs3 (< 0.001;0.78) ^*^ 2vs4 (0.001;0.91) ^*^ 2vs5 (0.025;1.19) ^*^ 3vs4 (0.995;0.09) 3vs5 (0.900;0.34) 4vs5 (0.970;0.28)
	Once a month (2)	79 (17.1)	0.91 (0.09)			76 (18.3)		
	2–4 times a month (3)	54 (11.7)	0.83 (0.19)			61.2 (20.1)		
	2–3 times a week (4)	36 (7.8)	0.78 (0.24)			59.4 (18.3)		
	4 or more times a week (5)	9 (2)	0.67 (0.30)			54.4 (17.4)		

^a^ TVT-Technical and Vocational Training

^b^ Unemployed, seasonal

**Table 2:** Summary score of HSUVs and EQ VAS by clinical characteristics (*n* = 462)

Clinical profiles		n (%)	EQ-5D-5 L			EQ VAS		
			Mean (SD)	*p-value*	*Scheffe p-value; Cohen’s d*	Mean (SD)	*p-value*	*Scheffe p-value; Cohen’s d*
Diagnosis	Anxiety (1)	149 (32.3)	0.88 (0.17)	0.005	1vs2 (0.851; -0.06) 1vs3 (0.049;0.30) ^*^ 2vs3 (0.009;0.35) ^*^	75.3 (17.9)	< 0.001	1vs2 (0.920;0.05) 1vs3 (< 0.001;0.61) ^*^ 2vs3 (< 0.001;0.53) ^*^
	Depression (2)	159 (34.4)	0.89 (0.18)			74.4 (19.7)		
	Both (3)	154 (33.3)	0.83 (0.16)			64.4 (17.9)		
Level of anxiety severity (GAD-7 scores)	Minimal (1)	-	-	< 0.001	2vs3 (< 0.001;0.52) ^*^ 2vs4 (< 0.001;0.82) ^*^ 3vs4 (0.185;0.21)	-	< 0.001	2vs3 (0.002;0.39) ^*^ 2vs4 (< 0.001;0.64) ^*^ 3vs4 (0.149;0.22)
	Mild (2)	173 (37.45)	0.93 (0.10)			76.8 (16.9)		
	Moderate (3)	185 (40.04)	0.85 (0.19)			69.7 (19.6)		
	Severe (4)	104 (22.51)	0.81 (0.20)			65.3 (19.5)		
Types of anxiety disorder	No anxiety disorder (1)	159 (34)	0.89 (0.18)	< 0.001	1vs2 (0.580;0.064) 1vs3 (< 0.001;0.72) ^*^ 2vs3 (< 0.001;0.82) ^*^	74.4 (19.7)	< 0.001	1vs2 (0.218;0.18) 1vs3 (0.001;0.63) ^*^ 2vs3 (0.013;0.49) ^*^
	Generalised anxiety disorder (2)	259 (56)	0.88 (0.14)			71.1 (18.3)		
	Others^a^ (3)	44 (10)	0.75 (0.24)			62 (18.9)		
Level of depression severity (PHQ-9 scores)	Minimal (1)	-	-	< 0.001	2vs3 (0.199;0.45) 2vs4 (< 0.001;0.78) ^*^ 2vs5 (< 0.001;1.1) ^*^ 3vs4 (0.001;0.55) ^*^ 3vs5 (< 0.001;0.85) ^*^ 4vs5 (0.316;0.18)	-	< 0.001	2vs3 (< 0.001;0.64) ^*^ 2vs4 (< 0.001;0.82) ^*^ 2vs5 (< 0.001;1.36) ^*^ 3vs4 (0.364;0.22) 3vs5 (< 0.001;0.66) ^*^ 4vs5 (0.023;0.39) ^*^
	Mild (2)	109 (23.59)	0.95 (0.07)			82.6 (13.8)		
	Moderate (3)	155 (33.55)	0.91 (0.10)			72.4 (17.4)		
	Moderately severe (4)	97 (21)	0.82 (0.23)			68.3 (20.8)		
	Severe (5)	101(21.86)	0.78 (0.21)			60.6 (18.3)		
Type of depressive disorder	No depressive disorder (1)	149 (32.3)	0.88 (0.17)	0.555	1vs2 (0.559;0.12) 1vs3 (0.989;0) 2vs3 (0.970; -0.12)	75.3 (17.9)	< 0.001	1vs2 (0.011;0.30) ^*^ 1vs3 (0.458;0.38) 2vs3 (0.985;0.05)
	Major depressive disorder (2)	299 (64.7)	0.86 (0.17)			69.5 (19.6)		
	Others^b^(3)	14 (3)	0.88 (0.11)			68.6 (16.9)		
Duration of illness (in years)	< 5 (1)	343 (74.2)	0.87 (0.16)	0.08	1vs2 (0.811; -0.125) 1vs3 (0.120;0.29) 2vs3 (0.102;0.35)	71.9 (18.4)	0.02	1vs2 (0.776; -0.10) 1vs3 (0.041;0.39) ^*^ 2vs3 (0.037;0.44) ^*^
	5–10 (2)	69 (15)	0.89 (0.16)			73.7 (20.1)		
	> 10 (3)	50 (10.8)	0.82 (0.24)			64.6 (21.4)		
Co-morbid medical diagnosis	No comorbid diagnosis (1)	391 (84.6)	0.88 (0.16)	0.02	1vs2 (0.954;0.12) 1vs3 (0.035;0.59) ^*^ 1vs4 (0.654;0.31) 2vs3 (0.380;0.34) 2vs4 (0.950;0.16) 3vs4 (0.776; -0.20)	72.3 (18.7)	0.02	1vs2 (0.967;0.10) 1vs3 (0.024;0.65) ^*^ 1vs4 (0.750;0.25) 2vs3 (0.304;0.48) 2vs4 (0.969;0.13) 3vs4 (0.649; -0.39)
	DM (2)	26 (5.6)	0.86 (0.17)			70.4 (22.9)		
	HTN^c^ (3)	25 (5.4)	0.78 (0.29)			60.2 (18.9)		
	Others^d^ (4)	20 (4.3)	0.83 (0.20)			67.6 (18.7)		
Medications for mental illness	Antidepressant (1)	242 (52.4)	0.89 (0.15)	0.02	1vs2 (0.022;0.30) ^*^ 1vs3 (0.989;0.06) 2vs3 (0.391; -0.21)	73.1 (18.7)	0.01	1vs2 (0.028;0.26) ^*^ 1vs3 (0.633; -0.16) 2vs3 (0.058; -0.43)
	Multiple (2)	181 (39.2)	0.84 (0.19)			68.1 (19.2)		
	Others^e^ (3)	37(8)	0.88 (0.2)			76 (14.1)		

^a^Social phobia, agoraphobia, anxiety disorder due to another medical condition, post-traumatic stress disorder

^b^dysthymia, substance-induced depressive disorder, premenstrual dysphoric disorder

^c^HTN-Hypertension

^d^pneumonia, malaria, febrile illness, asthma, breast cancer; ^e^antipsychotic, sedative-hypnotic, mood stabilizer

### Patient-reported health problems on the EQ-5D-5 L

**Fig. 1:**
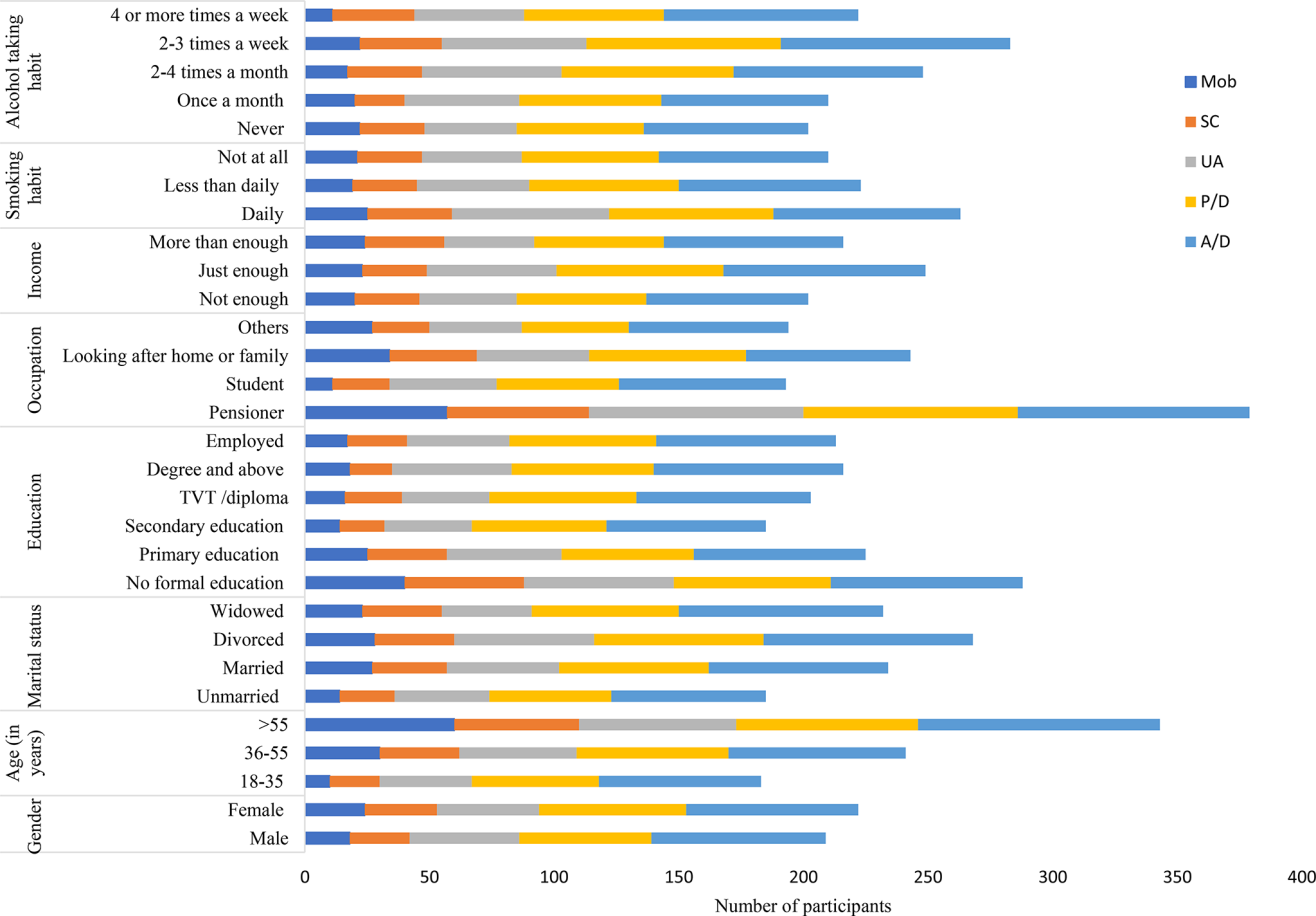
Number of participants (n) reported problems (levels 2–5) on EQ-5D-5 L health dimensions across socio-demographic and behavioural profiles (*n* = 462)

**Fig. 2:**
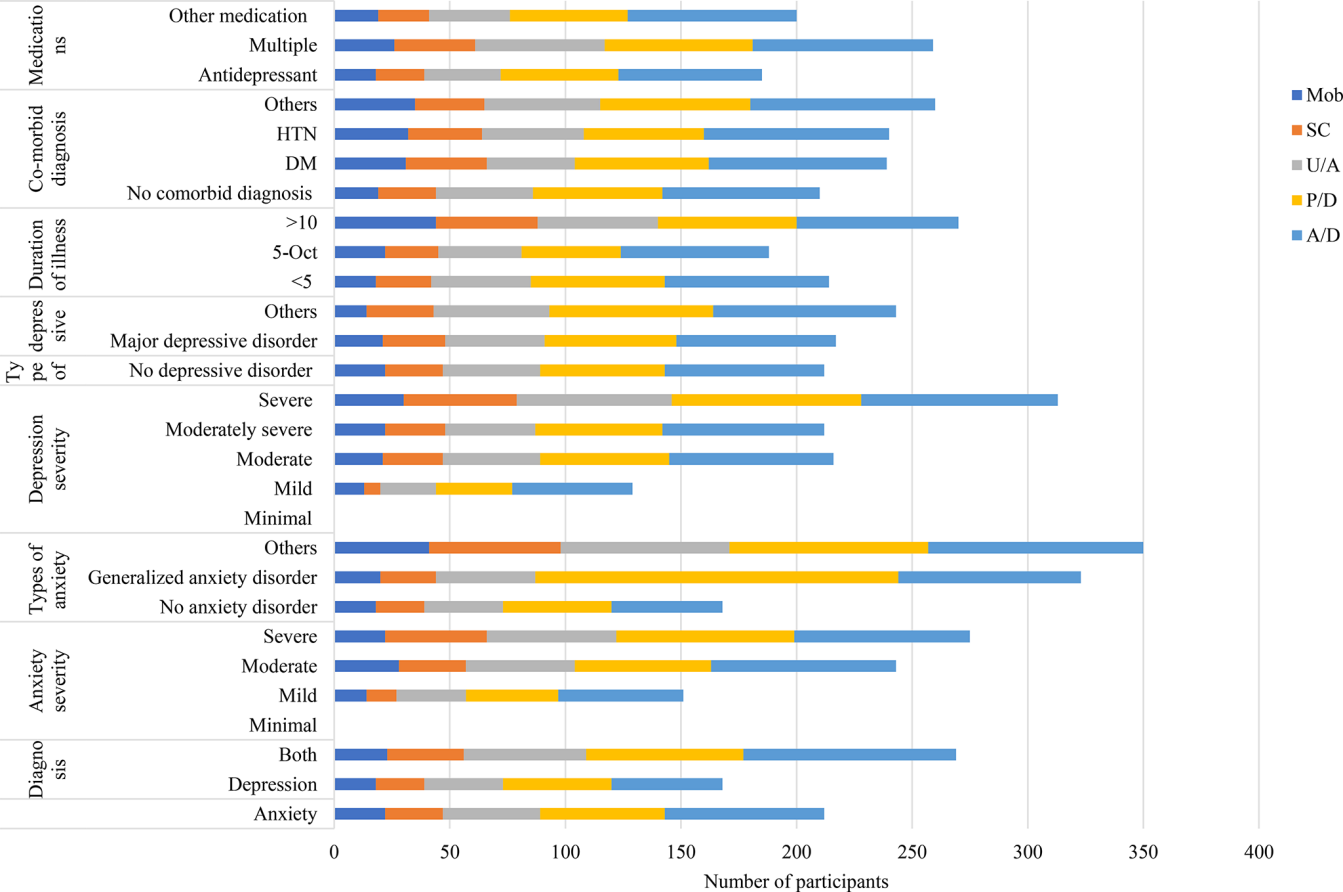
Number of participants (n) reported problems on EQ-5D-5 L health dimensions across clinical profiles (*n* = 462)

Regardless of differences in respondent demographic and clinical characteristics, a higher proportion of reported problems were observed in the P/D and A/D dimensions of the EQ-5D-5 L measure. The older age group and pensioners exhibited a higher prevalence of problems across various dimensions of the EQ-5D instrument (Fig. 1). Additionally, individuals with severe anxiety or depression symptoms reported more problems compared to those with milder symptoms (Fig. 2).

### **Patient-reported HSUVs and EQ VAS by respondent characteristics**

The PHQ-9, GAD-7 and EQ-5D-5 L demonstrated good internal consistency with a Cronbach’s alpha 0.90, 0.77 and 0.80 respectively. The mean HSUV and EQ VAS score of the total sample were 0.87 (SD = 0.17) and 71.4 (SD = 19.1), respectively. The age group of 18–35 years had the highest mean HSUVs (0.9) and EQ VAS score (73.8), while individuals over the age of 55 had the lowest mean scores for both HSUVs (0.78) and EQ VAS scores (61.8). The differences in reported HRQoL between the younger and older populations were large, as demonstrated by Cohen’s d effect sizes of 0.83 for HSUVs and 0.67 for EQ VAS scores (see Table 1). Patients experiencing both anxiety and depression scored significantly lower on HRQoL measures (HSUV = 0.83 [0.16], EQ VAS = 64.4 [17.9]) compared to those with either anxiety only (HSUV = 0.88 [0.17], EQ VAS = 75.3 [17.9]) or depression only (HSUV = 0.89 [0.18], EQ VAS 74.4 [19.7]). However, the observed effect sizes of these differences were small to moderate. Significant differences in the reported HSUVs and EQ VAS were observed across the various clinical variables, with the largest differences occurring between mild and severe conditions (see Table 2).

### **Patient-reported HSUVs**,** EQ VAS and associated respondents’ characteristics**

The results of the univariate analysis revealed significant negative associations between HSUVs and factors such as age (β = -0.003 [-0.004,-0.002]), GAD-7 score (β = -0.01 [-0.013, − 0.007]), PHQ-9 score (β = -0.009 [-0.011, − 0.007]), duration of illness (β = -0.004 [-0.007, − 0.001]), comorbid diagnosis (HTN: β =-0.103 [-0.172, − 0.034]), and multiple medication use (β = -0.047 [-0.079, − 0.014]). In contrast, having ‘more than enough’ income and being a non-smoker was associated with a higher score in HSUVs. Age was consistently and significantly associated with a lower HSUVs across the three models. The clinical profiles, including higher PHQ-9 scores, comorbid hypertension and multiple medication use, were also consistently negatively associated with the HSUVs across model 2 and model 3 (see Table 3).

**Table 3:** Regression analysis results with HSUVs as the outcome variable

Variables		Univariate β-Coeff (95% CI)	Multivariate β-Coeff (95% CI)					
			Model 1		Model 2		Model 3	
			β-Coeff	95% CI	β-Coeff	95% CI	Model3	95% CI
Gender(ref = Male)		-0.018 (-0.049, 013)	-0.025	(-0.059, 01)	-0.017	(-0.049, 0.015)	-0.028	(-0.062, 0.005)
Age (in years)		-0.003^*^ (-0.004, − 0.002)	-0.003^*^	(-0.005, − 0.001)	-0.002^*^	(-0.004, − 0.001)	-0.002^*^	(-0.004, − 0.001)
Marital status (ref = Unmarried)	Married	-0.012 (-0.047, 0.022)	-0.020	(-0.02, 0.06)	0.033	(-0.004, 0.068)	0.032	(-0.004, 0.068)
	Divorced	-0.060^*^ (-0.11, − 0.01)	-0.042	(-0.095, 0.012)	-0.029	(-0.085, 0.011)	-0.018	(-0.074, 0.025)
	Widowed	-0.045 (-0.12, 0.03)	0.007	(-0.073, 0.087)	0.032	(-0.042, 0.101)	0.033	(-0.042, 0.102)
Education (ref = No formal education)	Primary education	0.052 (-0.002, 0.106)	0.033	(-0.023, 0.089)	0.026	(-0.025, 0.077)	0.028	(-0.032, 0.069)
	Secondary education	0.061^*^ (007, 0.116)	0.026	(-0.034, 0.086)	0.018	(-0.04, 0.07)	0.018	(-0.041, 0.066)
	TVT /diploma^a^	0.061(-0.001 0.123)	0.042	(-0.025, 0.108)	0.027	(-0.03, 0.09)	0.034	(-0.038, 0.083)
	Degree and above	0.019 (-0.043, 0.082)	-0.007	(-0.077, 0.064)	-0.029	(-0.01, 0.04)	-0.023	(-0.09, 0.037)
Occupation (ref = Employed)	Pensioner	-0.13^*^ (-0.222, − 0.039)	-0.067	(-0.167, 0.032)	-0.08	(-0.17, 0.012)	-0.085	(-0.158, 0.022)
	Student	0.007 (-0.036, 0.05)	-0.042	(-0.093, 0.01)	-0.01	(-0.06, 0.04)	-0.004	(-0.066, 0.027)
	Looking after home or family	-0.032 (-0.079, 0.015)	-0.004	(-0.057, 0.049)	-0.01	(-0.06, 0.04)	0.011	(-0.047, 0.049)
	Others	0.011 (-0.033, 0.056)	0.004	(-0.044, 0.051)	-0.01	(-0.06, 0.03)	-0.008	(-0.054, 0.033)
Income (ref = Not enough)	Just enough	-0.041^*^ (-0.076, − 0.006)	-0.036	(-0.073, 0.001)	-0.025	(-0.06, 0.01)	-0.022	(-0.055, 0.012)
	More than enough	-0.033 (-0.103, 0.036)	-0.034	(-0.104, 0.036)	-0.036	(-0.01, 0.03)	-0.025	(-0.083, 0.047)
Diagnosis (ref = Anxiety)	Depression	0.011 (-0.027, 0.049)			0.08	(-0.02, 0.2)	0.071	(-0.036, 0.252)
	Both	-0.048^*^ (-0.086, − 0.01)			-0.01	(-0.09, 0.08)	-0.01	(-0.093, 0.141)
GAD-7 (Total score)		-0.010^*^ (-0.013, − 0.007)			-0.002	(-0.006, 0.002)	-0.002	(-0.005, 0.002)
Type of anxiety (ref = No anxiety)	Generalised anxiety disorder	-0.018 (-0.05, 0.015)			0.074^*^	(0.023, 0.125)	0.064	(-0.01, 0.157)
	Other anxiety disorder	-0.139^*^ (-0.2, − 0.08)			-		-	-
PHQ9 (Total score)		-0.009^*^ (-0.011, − 0.007)			-0.008^*^	(-0.01, − 0.005)	-0.008^*^	(-0.01, − 0.005)
Type of depressive disorder (ref = No depressive disorder)	Major depressive disorder	-0.019 (-0.052, 0.015)			-0.007	(-0.09, 0.08)	-0.005	(-0.141, 0.086)
	Other	-0.007 (-0.1, 0.09)			-		-	-
Duration of illness (in years)		-0.004^*^ (-0.007, − 0.001)			-0.001	(-0.004, 0.002)	-0.001	(-0.004, 0.003)
Comorbid (ref = No comorbid diagnosis)	DM	0.02 (-0.087, 0.048)			0.015	(-0.05, 0.08)	0.019	(-0.054, 0.07)
	HTN	-0.103 ^*^ (-0.172, − 0.034)			-0.08^*^	(-0.14, − 0.013)	-0.07^*^	(-0.134, − 0.006)
	Others	-0.05 (-0.126, 0.027)			-0.04	(-0.11, 0.031)	-0.04	(-0.113, 0.027)
Medication (ref = Antidepressant)	Multiple	-0.047^*^ (-0.079, − 0.014)			-0.044^*^	(-0.08, − 0.012)	-0.045^*^	(-0.08, − 0.018)
	Others	-0.004 (-0.06, 0.055)			-0.02	(-0.07, 0.04)	-0.021	(-0.215, 0.207)
Smoking habit (ref = Daily)	Less than daily	0.086^*^ (0.013, 0.158)					-0.014	(-0.083, 0.06)
	Not at all	0.091^*^ (0.03, 0.153)					0.02	(-0.038, 0.091)
Alcohol taking habit (ref = Never)	Once a month	0.024 (-0.015, 0.067)					0.024	(-0.019, 0.062)
	2–4 times a month	-0.051^*^ (-0.1, − 0.001)					0-0.008	(-0.047, 0.048)
	2–3 times a week	-0.104^*^ (-0.161, − 0.045)					-0.02	(-0.07, 0.052)
	4 or more times a week	-0.217^*^ (-0.325, − 0.105)					-0.124^*^	(-0.234, − 0.014)
R^2^			0.0844		0.2731		0.2905	
Adjusted R^2^			0.0536		0.2277		0.2356	
F			2.74^*^		6.01 ^*^		5.29^*^	

* p-value < 0.05

With regard to the EQ VAS, the univariate analysis presented in Table 4 demonstrate that being a non-smoker and having ‘more than enough’ income was associated to higher EQ VAS scores. Conversely, having a frequent alcohol drinking habit, a diagnosis of both anxiety and depression, a higher in PHQ-9 score, or having comorbid hypertension were associated with a decrease in EQ VAS scores. In the multivariate analysis, consistent positive associations with EQ VAS scores were observed for those who reported having enough income across all models (Model 3: β = 11.8 [4.5,19]). Clinical variables such as PHQ-9 score (Model 3: β = -0.87 [-1.16, -0.58]), having comorbid hypertension (Model 3: β = -7.14 [-14.28, -0.14]) and taking multiple medications (Model 3: β = -4.27 [-7.74, -0.8]) demonstrated an association with EQ VAS scores in both univariate and multivariate analyses.

**Table 4:** Regression analysis results with EQ VAS scores as the outcome variable

Variables		Univariate β-Coeff (*p*-value)	Multivariate β-Coeff (*p*-value)		
			Model1	Model2	Model3
Gender(ref = Male)		-3.46 ( -6.95, 0.029)	-2.94 (-6.85,0.98)	-1.84 (-5.41, 1.71)	-4.55^*^ (-8.3, − 0.81)
Age (in years)		-0.25^*^ (-0.398, − 0.095)	-0.27^*^ (-0.48, − 0.06)	-0.06 (-0.266,0.15)	-0.05 (-0.25, 0.16)
Marital status (ref = Unmarried)	Married	-1.21 (-5.02,2.61)	0.86 (-3.63,5.36)	1.15 (-2.94, 5.24)	1.23 (-2.76, 5.23)
	Divorced	-8.89^*^ (-14, -3.33)	-6.76^*^ (-13, − 0.71)	-6.29^*^ (-12, − 0.79)	-4.68 (-10, 0.8)
	Widowed	-9.27^*^ (-18, − 0.94)	-5.28 (-14, 3.71)	-4.25 (-12,3.9)	-3.29 (-11, 4.72)
Education (ref = No formal education)	Primary education	1.9 (-4.18,7.98)	1.38 (-4.91,7.66)	1.04 (-4.7, 6.77)	0.88 (-4.7, 6.5)
	Secondary education	5.5 (-0.61, 11.62)	3 (-3.71, 9.72)	2.88 (-3.23, 8.98)	2.82 (-3.15, 8.79)
	TVT /diploma^a^	4.64 (-2.25, 12)	2.77 (-4.69, 10.23)	1.71 (-5.13, 8.56)	2.95 (-3.77, 9.68)
	Degree and above	7 (-0.038,14)	3.38 (-4.51, 11)	1.3 (-5.91,8.51)	3.14 (-3.95, 10)
Occupation (ref = Employed)	Pensioner	-4.18 (-15,6.14)	1.85 (-9.36,13.06)	-0.95 (-11, 9.22)	-1.22 (-11.2, 8.76)
	Student	0.28 (-4.57,5.13)	-4.05 (-9.84,1.74)	-0.33 (-5.58,4.91)	-0.38 (-4.8, 5.56)
	Looking after home or family	-3.74 (-9.02,1.53)	1.21 (-4.76,7.19)	0.55 (-4.92, 6.02)	0.23 (-5.15, 5.6)
	Others	4 (-1,9|)	5.04 (-0.29,10.37)	3.35 (-1.54, 8.23)	3.72 (-1.1, 8.54)
Income (ref = Not enough)	Just enough	0.44 (-3.48, 4.37)	0.74 (-3.42 4.9)	2.48 (-1.3, 6.3)	2.55 (-1.2, 6.31)
	More than enough	9.72^*^ (1.93, 18)	10^*^ (2.21, 18)	10^*^ (3.18,17)	12^*^ (4.44, 19)
Diagnosis (ref = Anxiety)	Depression	-0.86 (-5.01,3.29)		-0.42 (-11, 12)	-0.15 (-11, 11)
	Both	-10.85^*^ (-15, -6.66)		-9 (-19, 0.77)	-7.51 (-17, 2.08)
GAD-7 (Total score)		-1^*^ (-1.36, − 0.64)		0.034 (-0.41, 0.48)	0.16 (-0.28, 0.6)
Type of anxiety (ref = No anxiety)	Generalised anxiety disorder	-3.32 (-7.06,0.41)		2.7 (-2.9, 8.33)	1.3 (-4.29, 6.9)
	Other anxiety disorder	-12.47^*^ (-19, -6.2)		-	-
PHQ9 (Total score)		-1.02^*^ (-1.24, − 0.8)		-0.95^*^ (-1.23, − 0.665)	-0.87^*^ (-1.16, − 0.58)
Type of depressive disorder (ref = No depressive disorder)	Major depressive disorder	-5.73^*^ (-9.47, -2)		-2.3 (-6.9, 11)	2.2 (-6.79, 11)
	Other	-6.64 (-17, 3.8)		-	-
Duration of illness (in years)		-0.48^*^ (-0.83, − 0.12)		-0.33 (-0.71, 0.047)	-0.36 (-0.73, 0.005)
Comorbid (ref = No comorbid diagnosis)	DM	-1.96 (-9.51, 5.6)		2.71 (-4.39, 9.824)	2.79 (-4.16, 9.73)
	HTN	-12^*^(-20, -4.41)		-9.12^*^ (-16, -1.9)	-7.14^*^ (-14.28, − 0.014)
	Others	-4.79 (-13, 3.76)		-3.51 (-11.4, 4.38)	-2.18 (-9.93, 5.56)
Medication (ref = Antidepressant)	Multiple	-5^*^ (-8.67, -1.34)		-4.26^*^ (-7.8, − 0.72)	-4.27^*^ (-7.74, − 0.8)
	Others	3.21 (-3.38,9.8)		0.73 (-5.56, 7)	0.025 (-6.13, 6.18)
Smoking habit (ref = Daily)	Less than daily	8.68^*^ (0.54,17)			0.91 (-7.07, 8.89)
	Not at all	9.71^*^ (2.83,17)			4.51 (-2.65, 12)
Alcohol taking habit (ref = Never)	Once a month	1.8 (-2.67, 6.44)			1.38 (-3.15, 5.92)
	2–4 times a month	-13^*^ (-18, -7.56)			-10^*^ (-15, -4.73)
	2–3 times a week	-15^*^ (-21, -8.33)			-7.04^*^ (-14, − 0.33)
	4 or more times a week	-20^*^ (-32, -7.51)			-12 (-24, 0.86)
R^2^			0.0783	0.2853	0.3311
Adjusted R^2^			0.0474	0.2406	0.2793
F			2.53 ^*^	6.39^*^	6.39^*^

* p-value < 0.05

## Discussion

### Main findings

The majority of respondents reported problems on the A/D (more than two thirds of respondents) and P/D (over half of the respondents) dimensions. The frequency of reported problems was higher among respondents experiencing more severity of anxiety or depression. This is consistent with a previous study, which reported that patients with mental illness, such as anxiety disorders, more frequently reported problems on the EQ-5D’s A/D and P/D dimensions compared to other dimensions [31]. The mobility and self-care dimensions had the least reported problems. However, it is important to note that the problems reported on mobility and selfcare should not be ignored. Adopting a comprehensive approach to treating anxiety and depression disorders that addresses both the psychological and physical aspects of the condition might improve the prevalence of reported problems across all the EQ-5D dimensions. Specifically, incorporating interventions that promote physical activity and self-care practices alongside treatments that primarily focus on patients’ mental health may lead to better outcomes in enhancing overall health outcome [32]. Our study findings indicate that older adults tend to report more problems across all five EQ-5D-5 L dimensions. This is supported by existing literature, which demonstrates a decrease in EQ-5D-5 L scores as individuals age increases [33].

The study results indicate that the reported mean score on the EQ-5D-5 L and EQ VAS were 0.87 (SD = 0.17) and 71.4 (SD = 19.1), respectively. These values were lower than the utility values of Ethiopia’s general population, which were 0.94 (SD = 0.10) and 87.26 (SD = 13.64) on the EQ-5D and EQ VAS scores, respectively [23]. The observed difference is expected since the general population includes both healthy individuals and those with disease conditions. However, the mean HSUVs reported by participants in the current study were lower than the values reported for patients with diabetes (mean EQ-5D-5 L = 0.95 and mean EQ VAS = 80) [34] and HIV/AIDS (mean EQ-5D-5 L = 0.95 and mean EQ VAS = 80) [35] in Ethiopia. Studies consistently indicate a minimum clinically important difference of 0.037 to 0.069 for the EQ-5D-5 L instrument [36, 37]. Therefore, in the context of chronic disease, the observed score differences hold significant clinical importance. The measurement scales used in this study demonstrated good internal consistency, with Cronbach’s alpha coefficients indicating strong reliability: PHQ-9 (α = 0.90) and GAD-7 (α = 0.77). These values are consistent with those previously reported in validation studies, where PHQ-9 had an alpha of 0.81 [24] and GAD-7 had an alpha of 0.77 [26].

The overall mean HSUV (Mean (SD) 0.87 (0.17) is surprisingly high compared to studies in high-income settings using the EQ-5D (mean (SD) 0.685 (0.218) for respondents with depression [16]. When looking at different levels of severity, our study found that the mean HSUVs for depression varied from 0.91 for moderate to 0.78 for to severe depression. In contrast, a study conducted in Sweden reported lower means ranging from 0.46 for moderate to 0.27 for severe depression [38]. This calls for a study to look at how people in high-income and low-income settings understand the EQ-5D questionnaire, which could potentially influence the instrument’s validity. Furthermore, the use of an interviewer administered version of the tool in our study may have introduced potential bias due to the presence of an interviewer, which may have led to positive responding bias.

The results suggest that self-reported health status may be influenced by both age and gender, with younger and male individuals reporting better health status. The literature also previously indicated that women are more likely than men to experience depression and that older adults are at a higher risk of developing mental health issues, including depression [39]. This highlights the potential need for targeted interventions to improve health outcomes for female and older populations.

In addition to comparing the mean scores, this study explored the association of individuals’ demographic, clinical and behavioural characteristics on the reported HSUVs and EQ VAS score. In the univariate analysis, a strong negative association was found between reported HSUVs and having an anxiety disorder (β=-0.139) or being a pensioner (β=-0.13), while a strong positive association was observed in non-smokers (β = 0.091) when compared to daily smokers. This observation is consistent with existing studies, which have shown that quitting smoking can have a substantial positive impact on reducing anxiety and depression, as well as improving overall quality of life [40] Therefore, it is imperative to emphasise and enhance behavioural education for smoking cessation, in line with the Ethiopian mental health policy [41].

A multiple regression analysis was used to investigate how various characteristics of the respondents are associated with the reported HSUVs and EQ VAS scores. Older age consistently showed a significant negative association with the HSUVs across all models. These findings highlight that it might be important to consider age, along with clinical and behavioural factors, when assessing the HSUVs. The pattern of association of variables with EQ VAS score showed some minor differences compared with the HSUVs. For instance, age showed significance only in the univariate analysis and Model 1 of the multiple regression analysis; however, it did not exhibit significance in Models 2 and 3. In addition to age, marital status and income were associated with EQ VAS scores. The discrepancy in results between the EQ VAS and EQ-5D-5 L may be attributed to the fact that these measures capture different concepts. The EQ VAS provides a subjective assessment of overall health using a single scale, while the EQ-5D-5 L encompasses multiple dimensions of HRQoL and assigns a value to different health states based on societal preferences [42].

Our study investigated the association between depression severity (PHQ-9), anxiety severity (GAD-7), and HSUVs as measured by the EQ-5D index. Despite the potential overlap between anxiety/depressive symptoms and health-related quality of life (HRQOL) measures, our primary objective was to establish HSUVs across varying levels of anxiety and depression severity. The PHQ-9 and GAD-7 are well-validated tools for assessing the severity of depression and anxiety, respectively, within the Ethiopian context [24, 26]. While these tools primarily focus on psychological well-being, the EQ-5D-5 L addresses multiple health dimensions, with only one item directly related to mental well-being (anxiety/depression). This led to some conceptual overlap due to shared constructs between the measures. This overlap might reflect a structural association, given the inclusion of mental health dimensions in both the PHQ-9/GAD-7 and the EQ-5D-5 L. Analytically, the correlation analysis revealed moderate correlations (*r* = -0.28 to -0.38) between the EQ-5–5 L and the symptom scales, aligning with previous studies that reported correlations in the range of *r* = -0.39 to -0.41, primarily driven by the anxiety/depression dimension of the EQ-5D-5 L [43]. These moderate correlations are consistent with the existing literature, suggesting that while some overlap exists, the tools also measure distinct constructs.

### Policy implications

This analysis has shown that older age or severity of the symptom and the presence of comorbidities, associated with a lower self-reported HRQoL, as depicted with reduced HSUVs and EQ VAS scores. However, marital status and income, as well as duration of illness and types of anxiety also have an association with HRQoL scores. Therefore, mental health service needs to be tailored to account for improvements in disease severity. Furthermore, social support packages that improve the income of individuals and counselling services related to marital or family issues might assist with improving the perceived HRQoL of patients. Income might be linked to access to healthcare, particularly in Ethiopia, where high out-of-pocket expenses are prevalent, potentially affecting patients’ HRQoL [44]. Our study has further important implications for the health technology assessment process in Ethiopia. The utility values reported in this study for various health states could be beneficial in conducting economic evaluations of anxiety and depression interventions in Ethiopia and similar resource-constrained settings.

## Strengths and limitations

This study is the first to establish HSUVs based on the severity of anxiety and depression and generate a coefficient based on symptom severity scores for Ethiopia. The study incorporated the preferences of the Ethiopian general population to estimate HSUVs for anxiety and depression [23]. This approach of estimating HSUVs offers an ethically acceptable foundation for resource allocation decisions, as it integrates societal preferences [7]. The inclusion of patients from different diagnostic groups and the use of a validated tool to assess symptom severity are also strengths of this study. However, there are some study limitations. While we observe variations in HSUVs based on the severity measured by the PHQ-9 and GAD-7, it is essential to recognise the conceptual overlap between these measures and EQ-5D-5 L particularly with the anxiety/depression dimension, which could contribute to this observed correlation [45]. The study’s applicability is limited to tertiary care service users and outpatients, as it did not include primary or secondary care service users or inpatients, in accordance with the country’s healthcare referral system. Participants were recruited using a convenience sampling approach within a single hospital. While this approach may have limited the generalisability of the study, it is important to note that the hospital from which participants were recruited is the only specialised psychiatric hospital in Ethiopia, representing the majority of patients affected by anxiety and/or depression. However, our findings should be interpreted within the context of this sampling approach, acknowledging the potential for selection bias. The reported literacy rate in our study was relatively higher (only 11% reported no formal education) compared to the low literacy rates in Ethiopia, with adult literacy at 52% [46]. Therefore, it is important to consider this limitation when extrapolating the findings within the country’s context. Administering the EQ-5D-5 L questionnaire through an interviewer may have introduced potential bias due to the presence of an interviewer, thereby limiting the applicability of the results to self-complete scenarios.

## Conclusion

In conclusion, this study found that overall, a higher proportion of reported problems was observed in the P/D and A/D dimensions of the EQ-5D-5 L measure. The age and clinical profiles of respondents were associated with differences in reported problems across EQ-5D-5 L health dimensions. The reported mean HSUVs and EQ VAS scores of respondents were lower than those reported in the general population and among individuals with other physical health conditions. Older age, higher levels of anxiety or depression severity and the presence of comorbid diagnoses were consistently associated with lower HSUVs and EQ VAS scores. To enhance health outcomes, clinicians managing anxiety or depression should consider age alongside clinical factors.

## Electronic supplementary material

Below is the link to the electronic supplementary material.

Supplementary Material 1

## Data Availability

The data supporting the findings of this study are available within the article and further information can be accessed from the first author (YBB) upon request.
